# An iterative approach for the global estimation of sentence similarity

**DOI:** 10.1371/journal.pone.0180885

**Published:** 2017-09-12

**Authors:** Tomoyuki Kajiwara, Danushka Bollegala, Yuichi Yoshida, Ken-ichi Kawarabayashi

**Affiliations:** 1 Tokyo Metropolitan University, Tokyo, Japan; 2 University of Liverpool, Liverpool, United Kingdom; 3 National Institute of Informatics, Tokyo, Japan; Institute of Microbial Technology CSIR, INDIA

## Abstract

Measuring the similarity between two sentences is often difficult due to their small lexical overlap. Instead of focusing on the sets of features in two given sentences between which we must measure similarity, we propose a sentence similarity method that considers two types of constraints that must be satisfied by *all* pairs of sentences in a given corpus. Namely, (a) if two sentences share many features in common, then it is likely that the remaining features in each sentence are also related, and (b) if two sentences contain many related features, then those two sentences are themselves similar. The two constraints are utilized in an iterative bootstrapping procedure that simultaneously updates both word and sentence similarity scores. Experimental results on SemEval 2015 Task 2 dataset show that the proposed iterative approach for measuring sentence semantic similarity is significantly better than the non-iterative counterparts.

## Introduction

Measuring the similarity between short textual units such as sentences, tweets or chat messages is a commonplace task in numerous natural language processing (NLP) applications such as information retrieval [1], text clustering, or classification [2–4]. Compared to measuring the similarity between longer textual units such as documents that contain many words, measuring the similarity between short sentences is a challenging task due to the lack of common features. Consequently, similarity measures based on word overlap such as cosine similarity, often fails to detect the similarity between sentences [5]. To overcome this feature sparseness problem, prior work on sentence similarity have proposed methods that use external lexical resources such as thesauri [6], or project sentences into a lower-dimensional dense spaces in which subsequently similarity is computed [7–12].

We propose a complementary approach for measuring the similarity between two sentences in a corpus that considers not only the features that occur in those two sentences, but also features that occur in *all* pairs of sentences in the corpus. Specifically, we require sentence similarity scores to satisfy two important types of constraints: (a) if two sentences share many common features, then it is likely that the remaining features in each sentence are also related, and (b) if two sentences contain many related features, then those two sentences are themselves similar.

To motivate the role played by these constraints consider the following three example sentences.

(i) *I love dogs and cats*.
(ii) *I love dogs and rabbits*.
(iii) *My favorite pet is a cat*.

Sentences (i) and (ii) share many common content words such as *I*, *love*, and *dog*. Thus, we can infer that *cat* and *rabbit* must also be semantically related. The confidence of our inference grows with (a) the proportion of the overlap, and (b) the number of different sentence pairs in which we observe similar overlaps. Consider now that we are further required to compare sentences (ii) and (iii), between which we have no common words. Without the knowledge that *cat* and *rabbit* are related from our previous comparison, we would predict a zero similarity score between sentences (ii) and (iii). However, if we use the knowledge obtained from (i) and (ii), and consider *cat* and *rabbit* to be similar (i.e. pets in this case), then we could predict a non-zero similarity score for (ii) and (iii). Therefore, we can benefit from the constraints derived from other pairs of sentences in a corpus (such as (i) and (ii)), when measuring the similarity between two given sentences selected from that corpus (such as (ii) and (iii)).

Our proposed method iterates over two stages.

- First, we align each sentence in a corpus with all the other similar sentences to build a word-alignment matrix. We compute the similarity between two words based on two factors: (a) pointwise mutual information between the two words according to their alignment frequency in the word-alignment matrix, and (b) prior similarity between words measured using pre-trained word embeddings. Using the computed word similarity scores, we measure the similarity between two sentences using three sentence alignment methods.
- Second, we update the word similarity scores using the word-alignment matrix computed in the first stage. Specifically, we propose two update rules for this purpose: an additive update, and a multiplicative update. The proposed method iterates multiple times over the corpus measuring similarities between all pairs of sentences. In practice, the proposed method converges in less than 3 iterations. However, computing all sentence pair similarities can be time consuming for large text corpora. To overcome this problem, we propose an efficient method to identify the top-most similar sentence pairs in a corpus that contribute to the similarity score update using SimHash [13] that obviates all-pair comparisons.

Our proposed method is unsupervised in the sense that it does not require any labeled data for sentence similarity. Moreover, we do not use external resources such as thesauri, which might not be available for resource poor languages or specialised domains. The proposed method does not assume a specific sentence representation method, and can be used with different sentence representations such as bag-of-words, or parse trees. Moreover, it is complementary to the sentence embedding methods, and can be used in conjunction in an ensemble setting as yet another sentence similarity measure.

We evaluate the proposed sentence similarity method using the SemEval-2015 Task 2 sentence similarity benchmark dataset. Our experimental results show that the proposed iterative approach for measuring sentence semantic similarity is significantly better than the non-iterative counterparts.

## Related work

Measuring the similarity between sentences is an omnipresent step in various NLP tasks such as paraphrase detection, recognizing textual entailment, sentence simplification and text summarisation.

In paraphrase detection, we must determine whether two sentences express the same meaning. Socher et al. [14] used recursive autoencoders to learn feature vectors for phrases. The feature vectors are then used to compute word- and phrase-wise similarity between sentences. A dynamic pooling layer is used to create a fixed-size representation for sentences of varying lengths. Finally, a supervised classifier is trained using this lower-dimensional embedding of sentences. Ji and Eisenstein [11] proposed a discriminative KL-divergence-based term weighting method and used matrix factorization to obtain lower-dimensional representations of sentences. Finally, a supervised classifier is trained using those sentence representation to detect similar sentence pairs. Cheng and Kartsaklis [15] used recursive neural networks for embedding a sentence in a latent dimensional space, in which similarity between sentences were measured. Representing sentences using latent features is an effective method to overcome the feature sparseness problem encountered when measuring the similarity between two sentences. Although we represented sentences using explicit lexical features, our proposed method *does not* depend on a particular sentence representation method, and can be applied with any of the representations proposed in prior work.

For recognising textual entailment, we must compare two sentences and decide whether one statement entails the other [16]. Sentence similarity measures have been used as features for recognizing entailment [17]. However, unlike similarity, entailment is an asymmetric relation [18]. In sentence simplification [19], for a given sentence, we must find a sentence that is simpler in terms of grammatical structure, word usage etc. than the original sentence. We believe that the word-alignment methods we propose in this paper will be useful for finding simplification candidates that preserve most information in the sentences to be simplified.

A benchmark dataset for sentence similarity was created via crowdsourcing in SemEval-2015 Task 2 [20]. Both supervised methods [21] that require sentence pairs annotated with similarity ratings, as well as unsupervised methods [22] have been proposed. Instead of using all the words in the two sentences, first selecting a subset of words from each sentence has been an effective technique [22–25]. Following this observation, we proposed maximum similarity and bipartite graph matching for selecting two subsets of words to be aligned between two sentences.

Pre-trained word embeddings have been successfully used in prior work to overcome feature spareness. Sultan et al. [23] used cosine similarity between word embeddings trained by CBOW [26] and lexical substitution features from PPDB [27] for measuring sentence similarity. Hänig et al. [24] used cosine similarity between word embeddings trained by SGNS [28] and features such as synonym from WordNet [29] and ConceptNet [30] for measuring sentence similarity. Han et al. [25] used cosine similarity between distributional word representations and features from WordNet for word-alignment. These best systems from the SemEval-2015 Task 2 are supervised methods or it depends on external resources. However, our proposed method is unsupervised and we do not use external resources. The main point in this paper is that the global sentence similarity computation method we propose can be used with *any* method for computing word similarity and representing a word/sentence embeddings.

An alternative method for measuring sentence similarity is to first embed each sentence into a space, and then measure cosine similarity in the embedded space. Skip-thought vector [31] and FastSent [32] are such sentence embedding methods that use consecutive triplets of sentences selected from books. In contrast to sentence embedding methods, our proposed method operates directly on pre-trained word embeddings to compute sentence similarity, without requiring us to learn sentence embeddings. This is particularly useful in situations where learning sentence embeddings is computationally expensive, or text corpora with sequential sentences are unavailable.

## Iterative similarity computation

Our proposed method iterates between two stages. First, we use the similarity between words to align pairs of sentences in a corpus. Following Song and Roth [22], we extend three sentence similarity measures for iterative similarity computation. Second, we update the word similarity scores considering the sentence alignments produced in the first stage. Two update rules are proposed for this purpose.

### Sentence alignment

Let us denote a sentence *x* by a vector $x=(x_{1},x_{2},\ldots ,x_{\left|V\right|})$, where the *i*-th element *x*_*i*_ is set to 1 if the *i*-th word occurs in the sentence *x*, and otherwise to 0. Here, vocabulary V is the set of words that occur in a corpus, and |V| denotes the number of unique words in that corpus.

Given a word-alignment method, A, the similarity, $S_{A}\left(x,y\right)$, between two sentences *x* and *y* can then be calculated using a word similarity measure *ϕ*(*x*_*i*_, *y*_*j*_). We use the following three word-alignment methods to define three sentence similarity measures.

#### Average similarity

The average similarity, *S*_*ave*_(*x*, *y*), between two sentences *x* and *y* is computed by averaging the similarities between all pairs of words taken from the two sentences as follows:

$$\begin{array}{c} S_{ave}\left(x,y\right)=\sum_{i=1}^{\left|V\right|}\sum_{j=1}^{\left|V\right|}\frac{x_{i}y_{j}\phi \left(x_{i},y_{j}\right)}{\left|\left|x\right|\right|\left|\left|y\right|\right|} \end{array}$$ (1)

Here, ||***x***|| denotes the ℓ_2_ norm of the vector ***x***. In particular, if *i* = *j* we set *ϕ*(*x*_*i*_, *y*_*j*_) = 1 and 0 otherwise, *S*_*ave*_ reduces to the popular cosine similarity.

#### Maximum similarity

Instead of averaging the word similarity scores, maximum similarity, *S*_*max*_(*x*, *y*), considers for each word *x*_*i*_ the most similar word *y*_*j*_, as follows:

$$\begin{array}{c} S_{max}\left(x,y\right)=\sum_{i=1}^{\left|V\right|}\frac{x_{i}\max_{j}y_{j}\phi \left(x_{i},y_{j}\right)}{\left|\left|x\right|\right|\left|\left|y\right|\right|} \end{array}$$ (2)

*S*_*max*_ can be considered as a sentence similarity measure based on a one-to-many word-alignment. We consider a word-pair (*x*_*i*_, *y*_*j*_) to be aligned if $j={\mathrm{argmax}}_{j^{\prime }}y_{j^{\prime }}\phi \left(x_{i},y_{j^{\prime }}\right)$. We create a word-alignment matrix **A**_*max*_ where the (*i*, *j*) element denotes the number of sentence pairs in which the *i*-th word of the first sentence was aligned with the *j*-th word of the second sentence.

#### Bipartite matching

We can represent the two sentences *x* and *y* by a bipartite graph where the vertices in each part correspond respectively to the two sets $\left\{i:i\in V,x_{i}=1\right\}$, and $\left\{j:j\in V,y_{j}=1\right\}$ consisting of words that occur in each sentence. Each vertex in the first part (corresponding to the words in the first sentence) is connected to all the vertices in the second part (corresponding to the words in the second sentence) using an undirected weighted edge. The weight of the edge connecting *i* to *j* is set to the word similarity *ϕ*(*x*_*i*_, *y*_*j*_). This bipartite graph can be constructed in $O(|V{|}^{2})$ time complexity.

Next, we can model the problem of measuring the similarity between the two sentences *x* and *y* as a problem of bipartite graph matching. Specifically, we would like to find the one-to-one mapping between the two parts that maximises the sum of edge-weights from *x* to *y*. Formally, let **M** be a boolean matrix where **M**_*i*,*j*_ = 1 if word *x*_*i*_ is aligned to word *y*_*j*_. Then the optimal word alignment has weight

$$\begin{array}{c} \max\sum_{i}\sum_{j}\phi \left(x_{i},y_{j}\right){\text{M}}_{i,j} \end{array}$$ (3)

such that each word *x*_*i*_ is aligned to at most one word *y*_*j*_. This maximum-matching problem can be solved using the Hungarian algorithm [33], a bipartite matching algorithm with time complexity $O(|V{|}^{3})$. For each word *x*_*i*_, let us denote its optimum alignment target under the Hungarian method by *y*_*j*_ = *y*_*h*(*i*)_.

$$\begin{array}{c} h\left(i\right)=\underset{j^{\prime }}{\mathrm{argmax}}{\text{M}}_{i,j^{\prime }} \end{array}$$ (4)

We define a similarity, *S*_*hun*_(*x*, *y*), based on this optimum alignment as follows:

$$\begin{array}{c} S_{hun}\left(x,y\right)=\sum_{i=1}^{\left|V\right|}\frac{x_{i}y_{h(i)}\phi \left(x_{i},y_{h(i)}\right)}{\left|\left|x\right|\right|\left|\left|y\right|\right|} \end{array}$$ (5)

*S*_*hun*_ can be considered as a sentence similarity measure based on a one-to-one word-alignment. We create a word-alignment matrix **A**_*hun*_ where the (*i*, *j*) element denotes the number of sentence pairs in which the *i*-th word of the first sentence was aligned with the *j*-th word of the second sentence according to the Hungarian algorithm.

### Incremental update rule

In many text similarity computation tasks such as finding similar documents in information retrieval, or document clustering, we must compare not only one pair of texts (documents) selected from a given collection, but compute the similarities between all pairs of texts. Likewise, when calculating the similarity between sentences, it is often the case that we are given a large collection of sentences (a corpus) from which a pair of sentences is selected. As we already described, we can exploit the information available in all the sentences in the corpus when measuring the similarity between two given sentences. Instead of considering the similarity between two words, *ϕ*(*x*_*i*_, *y*_*j*_), to be a fixed value, we update word similarities considering their alignments in sentences. Because the sentence similarity measures given by Eqs (1), (2) and (5) depend on the word similarity scores, this results in an update procedure that iterates between measuring sentence similarities (thereby word-alignments), and updating word similarity scores.

Let us denote the similarity between two words *x*_*i*_ and *y*_*j*_ after the *t*-th iteration by *ϕ*^(*t*)^(*x*_*i*_, *y*_*j*_), and the word-alignment matrix computed using the maximum similarity or the bipartite matching by **A**^(*t*)^. Note that the word-alignment matrix **A** is an asymmetric matrix. Therefore, we define a symmetric word co-occurrence matrix **C**^(*t*)^, where its (*i*, *j*)-th element is given by:

$$\begin{array}{c} C_{ij}^{\left(t\right)}=\left(A_{ij}^{\left(t\right)}+A_{ji}^{\left(t\right)}\right)/2 \end{array}$$ (6)

Let **B**^(*t*)^ be the word similarity matrix where its (*i*, *j*) element $B_{ij}^{\left(t\right)}$ denotes the similarity between the two words *i* and *j* computed using co-occurrence counts $C_{ij}^{\left(t\right)}$. Different word association measures can be used to compute similarity scores from co-occurrence counts. In this work, we use the positive pointwise mutual information (PPMI) [34] computed as follows:

$$\begin{array}{c} B_{ij}^{\left(t\right)}=\max\left(0,\log\left(\frac{C_{ij}^{\left(t\right)}\times \sum_{ij}C_{ij}^{\left(t\right)}}{\sum_{i}C_{ij}^{\left(t\right)}\sum_{j}C_{ij}^{\left(t\right)}}\right)\right) \end{array}$$ (7)

PPMI is frequently used for measuring word similarity in various NLP tasks [35].

We propose two update rules for updating the word similarity scores using the word-alignment counts: the *additive update rule* defined by Eq (8), and the *multiplicative update rule* defined by Eq (9).

$$\phi^{\left(t+1\right)}\left(x_{i},y_{j}\right)=\phi^{\left(t\right)}\left(x_{i},y_{j}\right)+\eta^{\left(t\right)}B_{ij}^{\left(t\right)}$$ (8)

$$\phi^{\left(t+1\right)}\left(x_{i},y_{j}\right)=\phi^{\left(t\right)}\left(x_{i},y_{j}\right)B_{ij}^{\left(t\right)}$$ (9)

Here, *η*^(*t*)^ is the update rate in the *t*-th iteration. Because we require word similarity scores to be in the range [0, 1], we scale *ϕ*^(*t*+1)^(*x*_*i*_, *y*_*j*_) by dividing from the maximum similarity score between any pair of words, max_*ij*_ *ϕ*^(*t*+1)^(*x*_*i*_, *y*_*j*_), after each iteration. In both update rules, the initial word similarities, *ϕ*^(0)^(*x*_*i*_, *y*_*j*_), are computed using pre-trained word embeddings. In our experiments, we used skip-gram with negative sampling (SGNS) [28] for learning word embeddings. Then, *ϕ*^(0)^(*x*_*i*_, *y*_*j*_) is computed as the cosine similarity between the word embeddings corresponding to the words *x*_*i*_ and *y*_*j*_.

The additive update rule given by Eq (8) closely resembles the update rule used in imitation learning [36], where a learner is required to imitate the training signal provided by an oracle. In our case, the word similarity scores *ϕ*^(*t*)^(*x*_*i*_, *y*_*j*_) are required to follow $B_{ij}^{\left(t\right)}$, the similarity scores computed using word-alignment counts. On the other hand, the multiplicative update rule given by Eq (9) can be seen as a weighted similarity score where current similarity scores are weighted by the corresponding alignment counts. We experimentally compare the different combinations of word-alignment matrices produced by different sentence similarity measures and the update rules.

In practice, even though two sentences might be similar, not all the words in the two sentences need to be similar. However, both maximum similarity method and the bipartite matching method require all word-pairs from the two sentences to be aligned. This imposes an unnecessarily strict constraint on word-alignment because two words might get aligned despite having a small word similarity score. To avoid such word-alignments, we consider only word-pairs (*x*_*i*_, *y*_*j*_) with similarity *ϕ*^(*t*)^(*x*_*i*_, *y*_*j*_) > *θ* for the word-alignment process for a fixed threshold *θ* ∈ [0, 1]. We experimentally study the effect of *θ* on the performance of our method.

### Efficient computation of similarity

Calculating the full word-alignment matrix requires computational complexity of $O(n^{2}|V|)$, where *n* is the total number of sentences in the corpus. However, most sentence pairs in a corpus will have almost zero similarity scores, and would not contribute to the word-alignment matrices. To avoid such unproductive computations, we use SimHash [13] to find the most similar *k* sentences for each sentence in the corpus, and measure sentence similarity only for those sentence pairs. Hamming distance over SimHash values of two sentences approximates the cosine similarity between the corresponding sentences. This method reduces the computational complexity to O(nk|V|), which is significantly smaller than $O(n^{2}|V|)$ for *k* ≪ *n*.

## Experiments

We evaluate the accuracy of our method by predicting the similarity between two given sentences using SemEval-2015 Task 2 sentence similarity benchmark dataset. Sensitivity of the performance for each parameter and initial word embeddings in our method is described.

### Sentence similarity measurement

For evaluating the proposed method for measuring sentence similarity, we use the SemEval-2015 Task 2 dataset (http://alt.qcri.org/semeval2015/task2/) [20]. This dataset includes 3,000 sentence pairs from five different domains: news headlines (Head), image descriptions (Img), answer pairs from a tutorial dialogue system (Stud), answer pairs from Q&A websites (QA), and sentence pairs from a committed belief dataset (Bel). Sentence similarity scores that range between 0 (the two sentences are completely dissimilar) to 5 (the two sentences are completely equivalent, as they mean the same thing) are obtained via crowdsourcing. A sentence similarity measure is evaluated against the human ratings in this dataset using the Pearson correlation coefficient. Pearson correlation coefficient ranges in [−1, 1], and high values indicate better agreement with the human notion of sentence similarity.

We use publicly available pre-trained word embeddings (https://code.google.com/archive/p/word2vec/) trained using SGNS and use cosine similarity to compute initial word similarities, *ϕ*^(0)^(*x*_*i*_, *y*_*j*_), required by the additive and the multiplicative rules defined respectively by Eqs (8) and (9). The pre-trained word embeddings are trained on about 100 billion word Google News corpus, and 300 dimensional vectors for 3 million words are created. We use 5-fold cross validation on the train sentence pairs in the SemEval-2015 Task 2 dataset to obtain the optimal values of *θ* = 0.4 and *t* = 3. Moreover, we experimented with different learning rate scheduling methods and found *η*^(*t*)^ = 1 to be the best. We analyse the sensitivity of the performance of the proposed method to those parameters. Because the SemEval-2015 Task 2 dataset contains only a small number of sentences (ca. 6,000), we do not require the SimHash-based approximation method for this dataset.

To demonstrate the effectiveness of conducting iterative similarity updates in the proposed method, we compare it against the following baseline methods that have been frequently used in prior work that do not perform iterative similarity updates.

Cosine baseline calculates the similarity between two sentences *x* and *y* as the cosine similarity between the two vectors *x* and *y* representing the two sentences.
Cosine (add SGNSs) baseline calculates the similarity between two sentences *x* and *y* as the cosine similarity between two sentence embeddings. These sentence embeddings are composed by adding the word embeddings of the words in each sentence. Representing sentences via the sum of word embeddings has been shown to be a strong baseline for creating sentence embeddings [32].
SGNS method calculates the similarity between two sentences *x* and *y* using the three sentence similarity measures, *S*_*ave*_, *S*_*max*_, and *S*_*hun*_ respectively using Eqs (1), (2) and (5). It uses the pre-trained word embeddings learnt using SGNS, and measures the similarity *ϕ*(*x*_*i*_, *y*_*j*_), between two words *x*_*i*_ and *y*_*j*_ as the cosine similarity between the corresponding word embeddings. This method simulates the proposals made by Song and Roth [22] for measuring sentence similarity using word alignments. This method *does not* perform any iterative similarity updates as done by the proposed method, and corresponds to the current state-of-the-art unsupervised sentence similarity measure.
PPMI baseline uses the PPMI-based word similarity computed using word-alignment counts, as the word similarity function *ϕ*(*x*_*i*_, *y*_*j*_), and computes the three sentence similarity measures *S*_*ave*_, *S*_*max*_, and *S*_*hun*_. Specifically, 6 variants of this baseline is computed by combining the two word-alignment matrices **A**_*max*_, and **A**_*hun*_, with the three sentence similarity measures *S*_*ave*_, *S*_*max*_, and *S*_*hun*_.

Table 1 compares the different sentence similarity measures using the Pearson correlation coefficients with the human ratings for the test sentence pairs in the SemEval-2015 Task 2 dataset. The proposed method (denoted by **Prop**) is computed for the combinations of 2 word-alignment matrices (**A**_*max*_ and **A**_*hun*_), 3 sentence similarity measures (*S*_*ave*_, *S*_*max*_, and *S*_*hun*_), and 2 update rules (additive and multiplicative, denoted respectively by + and *), resulting in 12 variants shown in Table 1. The final column, **Mean**, in Table 1 shows the weighted mean over the 5 domains for each method. It is computed by weighting the Pearson correlation coefficient in each domain by the total number of sentence pairs in that domain, according to the official scoring guidelines in SemEval-2015 Task 2.

**Table 1:** Sentence similarity measurement results on the SemEval-2015 Task 2 dataset.

Method	Head	Img	Stud	QA	Bel	Mean
Cosine	.531	.603	.664	.445	.651	.587
Cosine (add SGNSs)	.567	.531	.620	.296	.465	.525
SGNS *S*_*ave*_	.294	.316	.043	.079	.125	.189
SGNS *S*_*max*_	.603	.626	.656	.391	.636	.599
SGNS *S*_*hun*_	.590	.614	.682	.386	.615	.596
PPMI *A*_*max*_ *S*_*ave*_	.206	.325	.187	.236	.137	.226
PPMI *A*_*max*_ *S*_*max*_	.540	.561	**.701**	.327	.591	.565
PPMI *A*_*max*_ *S*_*hun*_	.531	.553	.697	.320	.574	.557
PPMI *A*_*hun*_ *S*_*ave*_	.340	.368	.327	.370	.221	.333
PPMI *A*_*hun*_ *S*_*max*_	.543	.602	.679	.437	.654	.592
PPMI *A*_*hun*_ *S*_*hun*_	.533	.586	.675	.430	.634	.582
Prop *A*_*max*_ + *S*_*ave*_	.456	.401	.374	.477	.255	.399
Prop *A*_*max*_ + *S*_*max*_	**.639**	**.643**	.674	**.501**	**.671**	.636*
Prop *A*_*max*_ + *S*_*hun*_	.626	.629	.674	.491	.654	.626
Prop *A*_*hun*_ + *S*_*ave*_	.443	.398	.361	.450	.254	.388
Prop *A*_*hun*_ + *S*_*max*_	.638	.642	.673	.498	.670	.634*
Prop *A*_*hun*_ + *S*_*hun*_	.626	.629	.674	.491	.654	.625
Prop *A*_*max*_ * *S*_*ave*_	.424	.395	.371	.444	.262	.386
Prop *A*_*max*_ * *S*_*max*_	.601	.631	.674	.480	.666	.620
Prop *A*_*max*_ * *S*_*hun*_	.591	.619	.674	.474	.650	.612
Prop *A*_*hun*_ * *S*_*ave*_	.423	.395	.370	.439	.262	.385
Prop *A*_*hun*_ * *S*_*max*_	.601	.631	.674	.479	.665	.619
Prop *A*_*hun*_ * *S*_*hun*_	.591	.619	.674	.474	.651	.612

The bold scores means the highest performance. The scores with a star statistically significantly outperform the SGNS (*S*_*max*_) baseline.

From Table 1, we see that **Prop** **A**_*max*_ + *S*_*max*_ is the best performing method among the different methods compared. In particular, it reports the best correlation coefficients in 4 out of the 5 domains. Moreover, according to the Fisher z-transformation, the correlations reported by the proposed method is statistically significantly better than that of **SGNS** *S*_*max*_, which supports our proposal that sentence similarities must be computed in an iterative fashion over the entire corpus considering word-alignment constraints. Overall, the maximum similarity word-alignment (**A**_*max*_) with *S*_*max*_ consistently perform well across different domains and baselines.

Between the two update rules, additive update outperforms the multiplicative counterpart. Recall that the word similarity matrix **B**^(*t*)^ given by Eq (7) is in practice a sparse matrix. Therefore, the multiplicative update rule given by Eq (9) results in even sparser similarity scores *ϕ*^(*t*+1)^ than *ϕ*^(*t*)^ after each update. On the other hand, the additive update rule given by Eq (8) would retain the non-zero elements in *ϕ*^(*t*)^ during the update. We believe that the extra sparsification in the multiplicative update rule decreases its performance when measuring the sentence similarities.

### Parameter sensitivity

We study the performance of the **Prop** **A**_*max*_ + *S*_*max*_ method, which reported the best results according to Table 1, under different update rate scheduling methods. Specifically, we consider update rate scheduling methods frequently used in stochastic optimization such as constant update rates (*η*^(*t*)^ = 0.5, 1.0, 1.5), reciprocal update rates (*η*^(*t*)^ = 1/*t*, 1/2*t*), and the inverse squared update rate (*η*^(*t*)^ = 1/*t*^2^).

**Fig 1:**
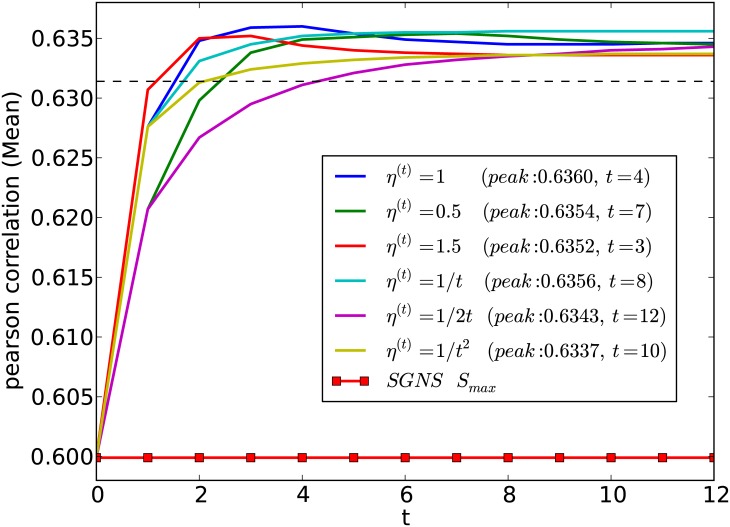
Effect of the different update rate scheduling methods on the performance of the proposed method is shown. The dashed horizontal line shows *p* < 0.05 significance level (Fisher z-transformation) for outperforming the **SGNS** *S*_*max*_ method. Peak correlation value and the required number of iterations (*t*) are shown within brackets.

Fig 1 shows the performance of the proposed method under different update rate scheduling methods. The dashed horizontal line in Fig 1 is the level of performance a particular method must obtain in order for that method to statistically significantly outperform the state-of-the-art **SGNS** *S*_*max*_. From Fig 1, we see that our proposed method outperforms **SGNS** *S*_*max*_ under all update rate scheduling methods. Therefore, the proposed method is relatively insensitive to the update rate scheduling method used.

Moreover, under constant update rates, when we increase the value of *η*, the Pearson correlation reaches the maximum value with a smaller number of iterations. Once the Pearson correlation coefficients have reached these maximum values, the performance converges. Because it is desirable to converge to the best correlation value with smaller number of iterations, *η*^(*t*)^ = 1.5 (peak performance achieved after 3 iteration) is a suitable value.

**Fig 2:**
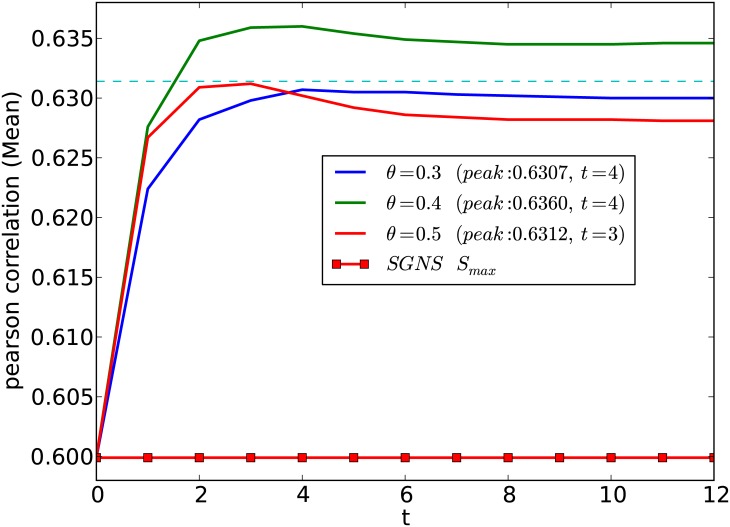
Effect of selecting word-pairs with similarity greater than *θ* for updating the word-alignment matrix. The dashed horizontal line shows *p* < 0.05 significance level (Fisher z-transformation) for outperforming the **SGNS** *S*_*max*_ method. Peak correlation value and the required number of iterations (*t*) are shown within brackets.

Fig 2 shows the effect of considering word-pairs greater than similarity *θ* during the sentence similarity measurement process. Considering less similar word-pairs in the alignment step leads to poor performance because of noisy alignments. On the other hand, high *θ* values will limit the number of words that we align between two sentences, leading to feature sparseness issues. This trade-off can be seen from the three curves shown in Fig 2.

**Fig 3:**
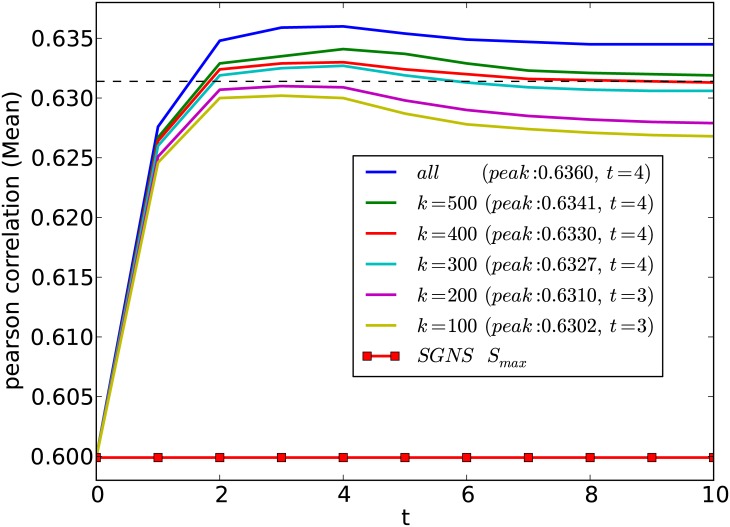
Effect of the number of top-*k* similar sentences selected using SimHash on the performance of the proposed method is shown. The dashed horizontal line shows *p* < 0.05 significance level (Fisher z-transformation) for outperforming the **SGNS** *S*_*max*_ method. Peak correlation value and the required number of iterations (*t*) are shown within brackets.

To study the effect of selecting top-*k* similar sentences using SimHash, in Fig 3 we measure the performance of **Prop** **A**_*max*_ + *S*_*max*_ against different *k* values. We see that even selecting a small sample as the top-most similar *k* = 100 sentences for each sentence in the corpus out of all sentences (ca. 6,000), the proposed method can obtain a high (0.6302) correlation coefficient. With *k* = 300 similar sentences we can obtain statistically significant improvements over **SGNS** *S*_*max*_. This is attractive when computing sentence similarities in large corpora. For example, even for a small corpus such as the SemEval-2015 Task 2 dataset, which has only 6,000 sentences, time taken for one iteration is reduced from 24 min to 1.5 min, by using *k* = 100.

**Fig 4:**
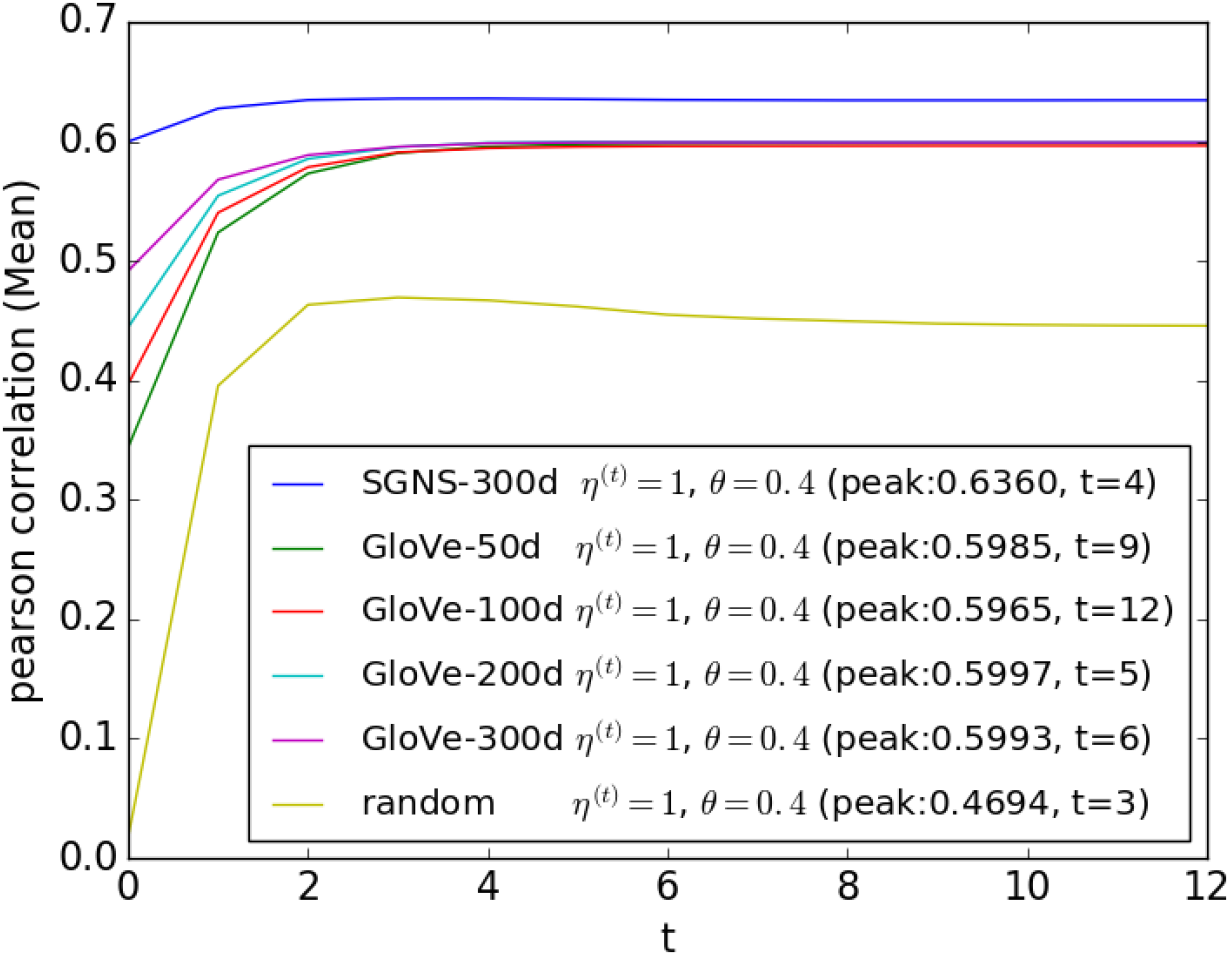
Effect of the different initial word embeddings on the performance of the proposed method is shown. The dashed horizontal line shows *p* < 0.05 significance level (Fisher z-transformation) for outperforming the **SGNS** *S*_*max*_ method. Peak correlation value and the required number of iterations (*t*) are shown within brackets.

To demonstrate the effect of the different initial word embeddings, we initialize using random vectors, and publicly available pre-trained word embeddings: 300 dimensional SGNS vectors (https://code.google.com/archive/p/word2vec/) for 3 million words, 50, 100, 200 and 300 dimensional GloVe vectors (http://nlp.stanford.edu/projects/glove/) for 400 thousand words. As shown in Fig 4, our proposed method can significantly improve any initial word similarity by iterative updating. The better performance of SGNS over GloVe can be explained by the larger vocabulary covered by SGNS.

### Sentence similarity complement

We improve an existing sentence similarity measure by a combination with the proposed method. The Word Mover’s Distance [37] which is a sentence similarity measure based on the dissimilarity between words is improved in this study.

Table 2 compares the different word dissimilarity measure for the Word Mover’s Distance. **Euclidean** baseline is calculated by the Euclidean distance ||***x***_*i*_ − ***y***_*j*_|| between word *x*_*i*_ and word *y*_*j*_ in the SGNS embeddings. **Prop** dissimilarity measure is calculated using our updated word similarity 1 − *ϕ*^(*t*)^(*x*_*i*_, *y*_*j*_). From Table 2, we can see that **Prop** method calculated using our updated word similarity improves Word Mover’s Distance [37] calculated using **Euclidean** distance. We confirmed the improvement of performance even in a small dataset (QA) consisting only of 375 sentence pairs.

**Table 2:** Sentence similarity results using Word Mover’s Distance on the SemEval-2015 Task 2 dataset.

Method	Head	Img	Stud	QA	Bel	Mean
Euclidean	.648	**.607**	.689	.428	**.552**	.609
Prop (t = 0)	.635	.588	**.702**	.477	.520	.606
Prop (t = 1)	**.651**	.592	**.702**	.495	.539	**.615**
Prop (t = 2)	**.651**	.592	.698	**.496**	.544	**.615**
Prop (t = 3)	.649	.593	.695	**.496**	.545	.614

## Conclusion

We proposed an unsupervised method to measure the similarity between two sentences which updates both word and sentence similarity scores in an iterative manner, making multiple passes over the entire corpus. Experimental results showed the effectiveness of the proposed iterative approach for measuring sentence semantic similarity. In future, we plan to apply the proposed method in large-scale paraphrase identification where we must detect similar sentence pairs among potentially large number of dissimilar sentence pairs.
